# Asymmetric Introgressive Hybridization Among Louisiana Iris Species

**DOI:** 10.3390/genes1010009

**Published:** 2010-03-15

**Authors:** Michael L. Arnold, Shunxue Tang, Steven J. Knapp, Noland H. Martin

**Affiliations:** 1 Department of Genetics, Life Sciences Building, University of Georgia, Athens, GA 30602, USA; 2 Trait Genetics and Technologies, Dow AgroSciences LLC, 9330 Zionsville Road, Indianapolis, IN 46268, USA; E-Mail: stang3@dow.com; 3 Monsanto Vegetable Seeds, 37437 California Highway 16, Woodland, CA 95695, USA; E-Mail: steve.knapp@monsanto.com; 4 Department of Biology, Texas State University – San Marcos, San Marcos, TX 78666, USA; E-Mail: nm14@txstate.edu

**Keywords:** asymmetric introgressive hybridization, Louisiana Irises, segregation distortion, natural hybrid zones

## Abstract

In this review, we discuss findings from studies carried out over the past 20+ years that document the occurrence of asymmetric introgressive hybridization in a plant clade. In particular, analyses of natural and experimental hybridization have demonstrated the consistent introgression of genes from *Iris fulva* into both *Iris brevicaulis* and *Iris hexagona*. Furthermore, our analyses have detected certain prezygotic and postzygotic barriers to reproduction that appear to contribute to the asymmetric introgression. Finally, our studies have determined that a portion of the genes transferred apparently affects adaptive traits.

## 1. Introduction

The network of interactions between flowering plants and their pollinators can be complex in terms of the number of pollinator classes visiting a given plant species. An added, and evolutionarily important, consequence can occur when there is spatial overlap between closely related plant taxa. Specifically, these co-occurrences may lead to the process of introgressive hybridization (or introgression), a process by which genes are transferred through the formation of an initial F_1_ hybrid that subsequently crosses with individuals of one or both of the parental species [1]. Some of the possible outcomes from introgression include: 1) the transfer of adaptive traits between the hybridizing lineages, 2) the formation of hybrid taxa (e.g., subspecies or species) and/or 3) the loss of one of the parental forms through genetic assimilation by the other, e.g., [2,3,4,5,6,7].

A pattern often seen in instances of introgression in natural populations is the asymmetric transfer of genetic material, see [6,9,10]. Thus, one of the hybridizing lineages acts mainly as a donor and the other taxon as a recipient of the genetic material in the transfer event. Some of the causal factors that have been suggested for asymmetric introgression include divergence among components of mating systems and ecological selection, e.g., [11,12].

Though numerous studies have detected asymmetric introgressive hybridization in nature, there are few examples in which multiple components of reproductive isolation (that may contribute to the pattern of asymmetry) have also been determined. In this paper, we review an example of consistent asymmetric introgression among species belonging to the Louisiana Iris plant assemblage. In particular, we will discuss genetic analyses of natural and experimental hybrid populations formed from crosses between *Iris fulva, Iris brevicaulis* and *Iris hexagona* that 1) detected the occurrence of asymmetric introgression, 2) defined some of the reproductive isolating barriers contributing to the asymmetric introgression and 3) revealed the genetic architecture (in terms of the distribution of segregation distortion) associated with this asymmetric exchange.

## 2. Louisiana Irises and asymmetric introgression in natural hybrid zones

**Figure 1:**
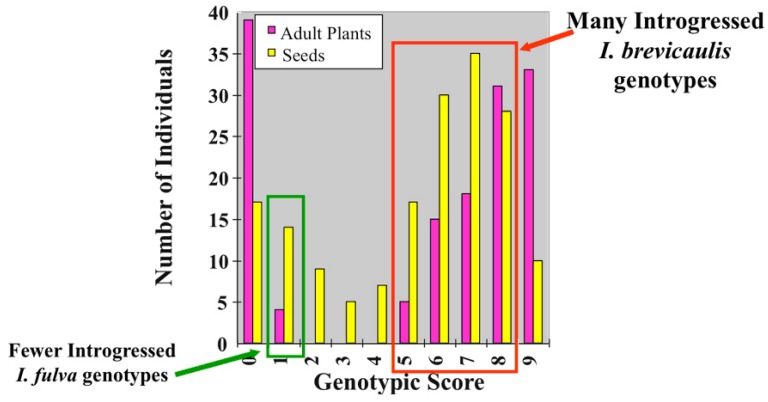
The distribution of genetic markers among adult plants and seeds collected from an *I. fulva* x *I. brevicaulis* natural hybrid population. The genotypic score was based upon nuclear and chloroplast DNA markers. Individuals with scores of “0” or “9” indicated *I. fulva* or *I. brevicaulis* individuals, respectively. Those adult plants or seeds with scores from 1-8 were hybrids [20]

Natural hybrid zones between the various species of Louisiana Irises have been documented since the first half of the 20th Century, e.g., [13,14,15,16,17,18,19]. A common observation in many of these studies has been asymmetry in introgression, with the transfer of more genic material from *I. fulva* into either *I. brevicaulis* or *I. hexagona*, than this species has received from either of the latter taxa. Figure 1 illustrates this class of observation for an *I. fulva* x *I. brevicaulis* hybrid population in southern Louisiana. The genetic markers used to define the various genotypic classes derived from both the chloroplast and nuclear genomes of these plants. Such combinations of cytoplasmic and nuclear data not only allow a cumulative genetic score to be obtained, but also provide estimates of cytonuclear incompatibilities, and are a standard measure for estimating genotypic diversity in natural hybrid zones (e.g., 5-7).

A significant proportion of the hybrid seeds and adult plants fell within the introgressed *I. brevicaulis* category (Figure 1). This indicates that gene flow (*i.e.,* introgression) occurred from *I. fulva* genomes into those of *I. brevicaulis*. In comparison, the reverse flow occurred much less frequently, reflected by the significantly fewer hybrids categorized as introgressed *I. fulva* [20]. An additional indication of the strong directionality was detected when single loci were analyzed. Table 1 presents one such nuclear locus. In particular, only 2.5% of the “*I. fulva*-like” plants and seeds were introgressed with *I. brevicaulis* alleles. In contrast, 72% of the *I. brevicaulis*-like plants possessed alleles introgressed from *I. fulva*. This highly significant difference in frequency of introgression [20] reflects well the extreme asymmetry in the pattern of gene flow between these two species.

**Table 1:** The frequency of homozygous *I. brevicaulis* (“bb”), heterozygous (“bf”) and homozygous *I. fulva* (“ff”) genotypes among adult plants and seeds from an *I. fulva* x *I. brevicaulis* natural hybrid zone [20].

	Adult Plant Genotypes			Seed Genotypes		
	*bb*	*bf*	*ff*	*bb*	*bf*	*ff*
*Iris brevicaulis*-like	17	20	0	29	89	9
*Iris fulva*-like	0	0	37	0	2	42

The pattern of *I. fulva >> I. brevicaulis* introgressive hybridization has been substantiated by studies of additional, natural hybrid zones. Arnold [21] and Johnston *et al.* [19] thus found a majority of hybrids in two separate hybrid zones to consist of *I. brevicaulis* genomes introgressed with *I. fulva* alleles. Importantly, Arnold [21] also detected a small number of hybrids in one hybrid zone that possessed *I. hexagona* genomes introgressed with *I. fulva* alleles as well. These latter data suggested that introgression between *I. fulva* and *I. hexagona* might have demonstrated a pattern of biased introgression from the former into the latter. In the following sections, we extend our discussion to studies of experimental *I. fulva* x *I. brevicaulis* and *I. fulva* x *I. hexagona* hybrid zones. Like the analyses discussed above, the findings from the experimental populations allow inferences concerning the directionality and extent of asymmetric introgression.

## 3. Louisiana Irises and asymmetric introgression: experimental hybrid populations

### 3.1. I. fulva x I. brevicaulis

**Figure 2:**
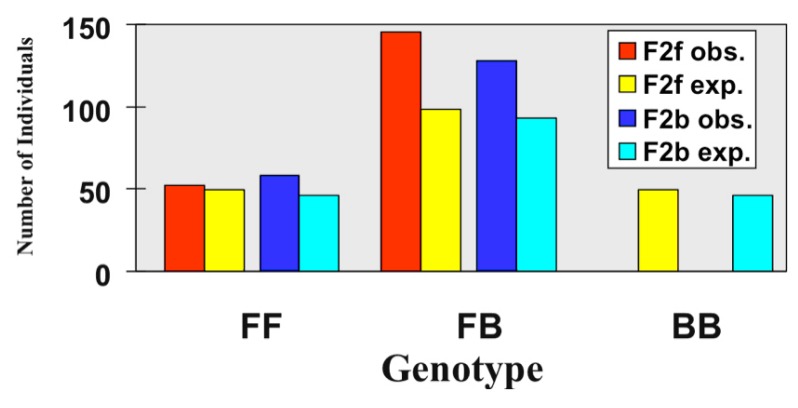
The observed and expected genotypic distributions at the L180 RAPD locus in *I. fulva* x *I. brevicaulis* F_2_ progeny derived from crosses with either *I. fulva* (“F2f”) or *I. brevicaulis* (“F2b”) as the female parent [23].

It is now well-recognized that hybridization can give rise to not only less fit genotypes, but also hybrid genotypes that, in certain environments, demonstrate elevated fitness relative to their parents (e.g., see [6-8,22]). In regard to the present discussion, such differential selection on hybrid genotypes could contribute to asymmetric introgression. Figure 2 illustrates the results of a test for differential selection among *I. fulva* x *I. brevicaulis* F_2_ genotypes at a single locus. The pattern of expected and observed frequencies of the three possible genotypes (homozygous for the *I. fulva* or *I. brevicaulis* alleles or heterozygous for these alleles) did indeed support a role for differential selection in the production of asymmetric introgression in this species pair. Specifically, the homozygous *I. brevicaulis* genotype was absent. Thus, there were significantly less than expected *I. brevicaulis* alleles in the F_2_ hybrids, and a significantly greater than expected frequency of *I. fulva* alleles [23]. As with the findings for the single locus sampled in the natural hybrid population (Table 1), the presence of significantly more *I. fulva* than *I. brevicaulis* alleles in the F_2_ hybrid progeny (Figure 2), is consistent with a greater level of introgression from the former species into the latter.

**Figure 3:**
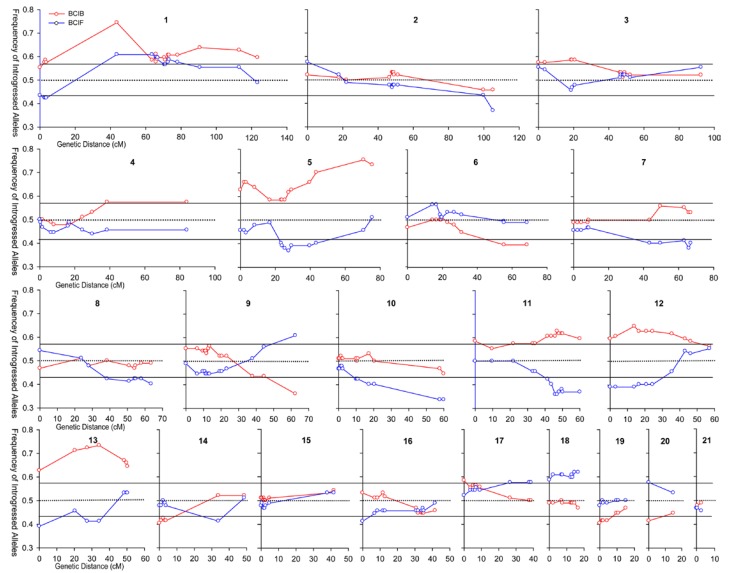
The observed frequencies of introgressed alleles from either *I. fulva* (red lines) or *I. brevicaulis* (blue lines) into first generation backcross progeny formed from crosses between these two species. The X-axis indicates the genetic distances (in centimorgans) along each of the 21 linkage groups in the composite map. The Y-axis indicates the transmission ratio of either the *I. fulva* alleles or *I. brevicaulis* alleles introgressed into the backcrosses toward the alternate species. The expected frequency is 0.50 and is indicated by the dotted line. Data points above and below the solid lines indicate significant deviations from 0.50 (α = 0.05). Frequencies > 0.50 indicate an overrepresentation of either the *I. fulva* (red line) or *I. brevicaulis* (blue line) alleles in the genetic background of the alternate species. Frequencies < 0.50 indicate an underrepresentation of these same categories [24].

As with the analyses of allele frequencies at individual loci (Table 1 and Figure 2), whole-genome scans have likewise detected asymmetric transfer of genetic material between *I. fulva* and *I. brevicaulis*. Figure 3 illustrates the findings from a linkage map analysis using hybrid individuals from two first-generation backcross populations; one population was constructed by crossing an F_1_ plant with *I. fulva* and the other through crosses between an F_1_ individual and *I. brevicaulis*. Approximately 1/3 of the genetic markers occurred at either significantly higher or lower frequencies than expected (*i.e.,* demonstrated “transmission ratio distortion”) in each of the reciprocal backcross maps (Figure 3; [24]). The distortion in transmission was biased, with *I. fulva* alleles largely overrepresented at the expense of *I. brevicaulis* alleles. Specifically, 18 separate regions demonstrated significant introgression of *I. fulva* alleles into the *I. brevicaulis* genetic background (*i.e.,* in the backcross hybrids towards *I. brevicaulis*). Furthermore, 12 regions in the backcross population towards *I. fulva* also showed elevated frequencies of *I. fulva* alleles. In contrast, *I. brevicaulis* alleles were significantly overrepresented in only five locations in each of the backcross populations (Figure 3).

This pattern of transmission ratio distortion caused Tang *et al.* [24] to conclude the following: “Whatever the mechanism(s) involved, given that these two species hybridize in nature, this asymmetry in gene flow could have important implications for introgressive hybridization. Namely, we would expect that for a majority of the regions revealing transmission ratio distortion, *I. fulva* alleles might be favored to introgress into a predominately *I. brevicaulis* species-background, while the introgression of *I. brevicaulis* alleles into *I. fulva* would be retarded.”

### 3.2. I. fulva x I. hexagona

**Figure 4:**
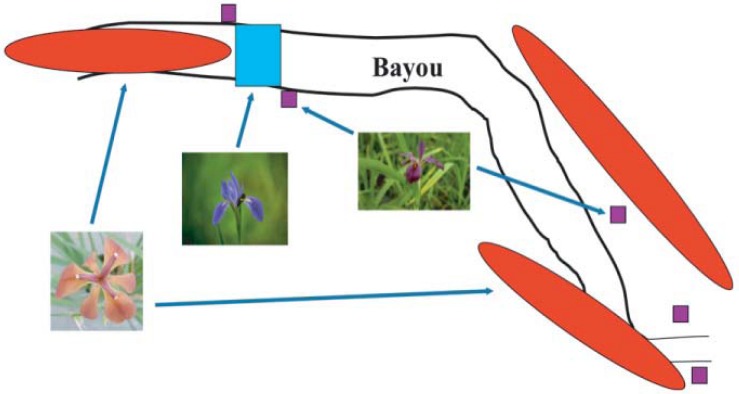
Schematic illustration of the distribution of 1) naturally occurring *I. fulva* plants (red ovals), 2) introduced *I. hexagona* (blue rectangle) and 3) *I. fulva* x *I. hexagona* F_1_ plants (purple squares) [27,28].

Natural and experimental hybridization between *I. fulva* and *I. brevicaulis* consistently produces asymmetric introgression. One hypothesis derived from this observation is that asymmetric introgressive hybridization is only typical for reproductive interactions between these two species, and not other species of Louisiana Irises. It is possible to test this hypothesis using a series of genotyping assays carried out in an experimentally constructed iris population in southern Louisiana (Figure 4).

**Figure 5:**
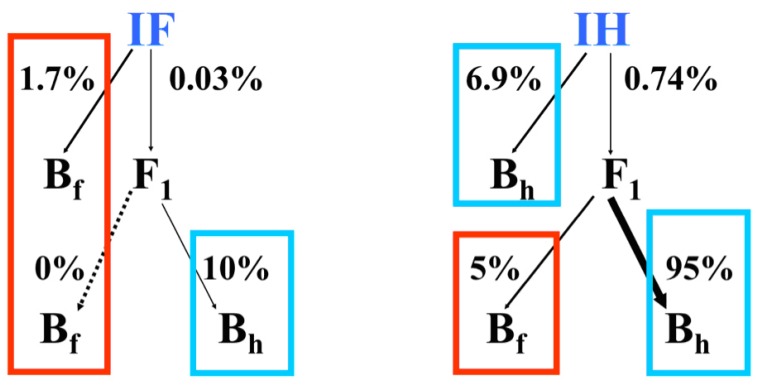
Percentage of F_1_ (0.03% and 0.74% in *I. fulva* and *I. hexagona* fruits, respectively) and first generation backcross seeds (B_f_ and B_h_) formed on plants in an experimental population by natural pollinations [27,28]. The B_f_ and B_h_ hybrid seeds reflect the first generation of introgression into *I. fulva* and *I. hexagona*, respectively.

The experimental population lies within a region typified by numerous natural hybrid zones between *I. fulva, I. brevicaulis* and *I. hexagona*, e.g., [16,25,26]. Originally, this population consisted only of naturally occurring *I. fulva* individuals. In 1989, we introduced 200 *I. hexagona* plants in a centralized block (Figure 4). Over three consecutive years, we collected and genotyped >5000 seeds from *I. fulva* and *I. hexagona* fruits formed by pollen transfer by natural pollinators. F_1_ hybrid seed formation was very infrequent in the fruits of both species. However, there was a significant bias in the direction of hybrid formation (Figure 5) with F_1_ seeds being formed at 50x the frequency in *I. hexagona* fruits (*i.e.,* 0.74%) relative to *I. fulva* fruits (*i.e.,* 0.03%; [27,28]).

The asymmetry in the frequency of F_1_ formation between *I. fulva* and *I. hexagona* – like hybrid formation in general being limited more in *I. fulva* fruits – is consistent with directional introgression from the former into the latter species. Yet, it should be kept in mind that, though F_1_ hybrid formation is requisite for introgression, this hybrid stage does not reflect an introgressed generation. The introduction of experimentally formed F_1_ hybrids into this same population did, however, allow a test for asymmetric introgression. In particular, we collected and genotyped seeds from fruits produced by *I. fulva*, *I. hexagona* and F_1_ plants (spatially near either *I. fulva* or *I. hexagona* plants; [28]). The determination of the frequencies of backcross hybrids toward the two species allowed another test for asymmetric introgression.

The placement of F_1_ plants near either *I. fulva* or *I. hexagona* individuals allowed an estimate of backcross formation in a spatial context (Figure 5). The frequencies of backcross seeds toward *I. fulva* were 1.7% and 0% in *I. fulva* and F_1_ fruits, respectively when F_1_ plants were spatially adjacent to *I. fulva* plants. Additionally, 5% of the seeds in F_1_ fruits from hybrid plants near *I. hexagona* possessed *I. fulva* backcross genotypes. In contrast, the frequencies of backcross hybrids toward *I. hexagona* were significantly greater, regardless of spatial arrangement of F_1_ plants. Thus, the frequency of backcross hybrids in F_1_ fruits from plants near either *I. fulva* or *I. hexagona* was 10% and 95%, respectively (Figure 5). Finally, first generation backcross seeds were formed at a frequency of ca. 7% in *I. hexagona* fruits. Just as with introgression between *I. fulva* and *I. brevicaulis*, strong asymmetry in the formation of both F_1_ and backcross hybrid progeny between *I. fulva* and *I. hexagona* was detected [28].

## 4. The causes of asymmetric introgression in Louisiana Irises: Prezygotic reproductive isolation

The Louisiana iris species complex has been recognized for decades as a paradigm for examining processes associated with natural hybridization and speciation [2,5,6,7]. Indeed, this complex is now recognized as a model system for describing a number of the possible outcomes of reticulate evolution [7], and the causal factors affecting the outcomes. In regard to the present discussion, it is possible to ask if prezygotic and/or postzygotic reproductive isolating barriers might contribute to the observed asymmetric introgressive hybridization between *I. fulva* and its two congeners.

**Figure 6:**
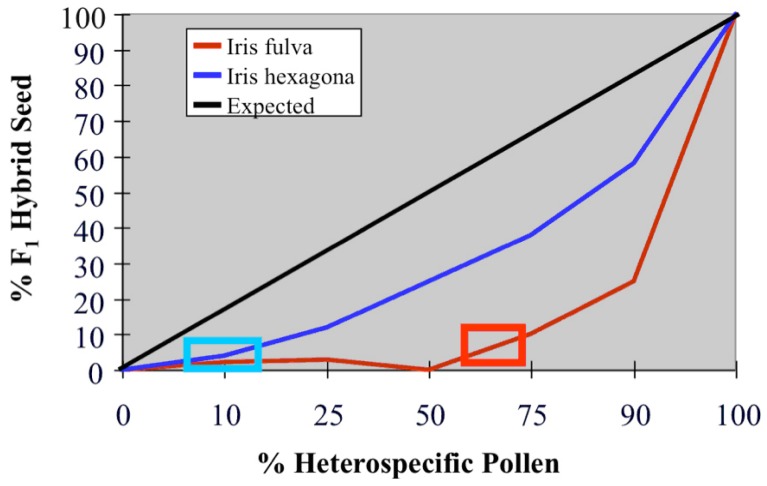
Percent F_1_ hybrid seeds produced by various mixtures of *I. fulva* and *I. hexagona* pollen. The solid line illustrates the expected percentage of hybrid seeds assuming random fertilization. All the observed F_1_ percentages were significantly less than expected (except for the 0% and 100% treatments, in which there were no mixtures of conspecific and heterospecific pollen). The blue and red rectangles indicate the percentage of heterospecific pollen necessary to increase significantly F_1_ hybrid formation above the value of “0” in *I. hexagona* and *I. fulva* fruits, respectively. Note the much greater frequency of F_1_s formed in *I. hexagona* fruits relative to *I. fulva* fruits [29].

One of the best-documented, prezygotic, barriers between these three species is gamete competition. Gamete competition is defined by the observation that when mixtures of conspecific and heterospecific sperm or pollen are made available for the fertilization of eggs, the conspecific gametes father more offspring than expected. An example of gamete competition between *I. fulva* and *I. hexagona* is illustrated in Figure 6. Carney *et al.* [29] detected a significant reduction in the frequency of hybrid seed formation for each of their pollination treatments involving mixtures of conspecific and heterospecific pollen (Figure 6). The pattern of hybrid seed formation indicated that post-pollination (but pre-fertilization) phenomena were limiting the formation of F_1_ hybrid seeds. As the proportion of heterospecific pollen in the mixtures increased, so did the proportion of hybrid seeds formed, albeit at a significantly lower frequency than expected. However, there was strong asymmetry in the degree of reproductive isolation, with F_1_ formation being much greater in the direction of *I. hexagona* than towards *I. fulva*. As mentioned previously, the F_1_ generation is not “introgressed”. However, if gamete competition continues to affect the formation of later generation hybrids, it could act as a causal factor in the observed asymmetric introgressive hybridization in natural hybrid zones between *I. fulva* and *I. hexagona*.

In addition to the findings of Carney *et al.* [29], Emms *et al.* [30] also detected asymmetric effects from gamete competition between *I. fulva* and *I. brevicaulis*. Pollen tube growth measurements led to the prediction that relatively more conspecific progeny would be produced by *I. fulva* than *I. brevicaulis* flowers, when both pollen types were present on the same stigma. This prediction was supported with results from a seed siring experiment. The application of 50% : 50% mixtures of *I. fulva* and *I. brevicaulis* pollen to the stigmas of both species resulted in 24.1% and 38.6% F_1_ hybrid seeds in *I. fulva* and *I. brevicaulis* fruits, respectively. These frequencies are significantly different from one another and from the expected 50% : 50% ratio [30], once again supportive of gamete competition playing a role in asymmetric introgression between these two iris species.

## 5. The causes of asymmetric introgression in Louisiana Irises: Postzygotic reproductive isolation

### 5.1. Selection at early life history stages

As discussed above, introgression occurs largely from *I. fulva* into *I. brevicaulis* and *I. hexagona* in both natural and experimental hybrid populations. Some of these results also allow a determination of possible reproductive barriers that contribute to this asymmetry. Gamete (or pollen) competition was presented as one of the prezygotic barriers underlying this asymmetry. There is also evidence that postzygotic barriers contribute to the directionality of introgression. In particular, viability selection disfavors introgressed genotypes towards *I. fulva*, but favors those towards *I. brevicaulis*.

Figures 1 and 2 and Table 1 illustrate the effects of natural selection, at the seedling establishment stage, against certain hybrid genotypes in both natural and experimental hybrid populations. For example, there are numerous *I. fulva*-like introgressed genotypes present in the seeds sampled from the natural hybrid zone that are not present in the adult iris plants (e.g. those with genotypic scores of 2-4; Figure 1). In contrast, there are adult plants in all of the *I. brevicaulis*-like introgressed categories (*i.e.,* 5-8). This indicates viability selection *against* introgressed *I. fulva* genotypes, but likely *for* certain introgressed *I. brevicaulis* genotypes [6,20]. Similarly, Figure 2 reflects selection that disfavors hybrids containing a higher frequency of introgressed *I. brevicaulis* alleles, but favors hybrids with a higher proportion of *I. fulva* alleles [23]. Finally, this lack of penetration of *I. brevicaulis* alleles can also be seen in the data presented in Table 1. In this instance, data from a single locus (as with those given in Figure 2) indicates that *I. fulva* alleles are incorporated into adult *I. brevicaulis*-like plants, but *I. brevicaulis* alleles are almost completely excluded from plants that are *I. fulva*-like [20].

### 5.2. Selection at later life history stages

From the above, we see that there is evidence consistent with selection-generated asymmetry in introgression due to differential viability at early life history stages in Louisiana Irises. Similarly, a number of analyses have detected asymmetry in survivorship at latter stages of plant development. For example, the segregation distortion illustrated by Figure 3 is due to differential survivorship of adult plants maintained in the greenhouse. Thus, even under what is assumed to be highly favorable environmental conditions (*i.e.,* the greenhouse), asymmetric introgression from *I. fulva* into *I. brevicaulis* was detected [24].

The pattern of survivorship under natural conditions has also been studied by transplanting the same genotypes maintained in the greenhouse [24] into field conditions in southern Louisiana. Table 2 contains the observed survivorship frequencies for *I. fulva*, *I. brevicaulis* and introgressed genotypes of both species subsequent to a severe (water depth of several feet) and extended (ca. four month) natural flooding event [31]. We detected the following, hierarchical, survivorship values: *I. fulva* > Introgressed *I. fulva* > Introgressed *I. brevicaulis* > *I. brevicaulis*. This pattern of survivorship is consistent with previous observations suggesting greater tolerance to root/rhizome submersion by *I. fulva* relative to *I. brevicaulis* [18]. 

**Table 2:** Survivorship frequencies for *I. brevicaulis*, *I. fulva* and introgressed genotypes of these two species. Survivorship estimates were derived after a severe flooding episode [31].

Class	Alive	Dead	% Survival
I. brevicaulis	0	13	0
Introgressed I. brevicaulis	23	393	0.055
Introgressed I. fulva	33	325	0.092
I. fulva	3	8	0.273

In regard to asymmetric introgression, it might be argued that the above pattern (of greater survivorship of Introgressed *I. fulva* genotypes relative to Introgressed *I. brevicaulis* genotypes) would facilitate greater levels of introgression into *I. fulva*. However, the data from both Table 2 and Table 3 argue against this inference. Instead, it is apparent that *I. fulva* alleles often (but, not always, see [31]) provide the basis for higher survivorship. First, the surviving “Introgressed *I. brevicaulis*” genotypes contained a significantly higher number of positively selected *I. fulva* alleles than *I. brevicaulis* alleles (Table 3, [31]). Second, *I. fulva* and “Introgressed *I. fulva*” hybrids survived at the highest frequencies (Table 2), reflecting the selective advantage of this species’ genetic background in a flooded environment. These field surveys will also allow an estimate of temporal fluctuations in the direction and/or strength of selection (e.g. as expected if drier environmental conditions, favoring *I. brevicaulis*, occur).

**Table 3:** The number of *I. fulva* and *I. brevicaulis* alleles inferred to be selectively favored in “Introgressed *I. brevicaulis*” plants that survived the flooding episode (see Table 2; [31]).

	Introgressed *I. brevicaulis*	
Alleles Favored	*I. fulva*	*I. brevicaulis*
Number of Alleles Favored	101	41

## 6. Asymmetric introgression and adaptive trait transfer

The studies discussed in this review provide evidence that selection causes at least a portion of the asymmetry in gene flow among the Louisiana Iris species. The available data also allow a test of whether or not some of the asymmetric introgression reflects adaptive exchanges: 1) the patterns of segregation distortion in the greenhouse-maintained plants (Figure 3), as well as survivorship under stress (*i.e.,* severe flooding), support the inference of adaptive transfers; and 2) the presence of *I. fulva* alleles allowed introgressed individuals to survive and persist at a significantly higher frequency than those plants with fewer (or no) *I. fulva* alleles, under a variety of environmental conditions.

**Figure 7:**
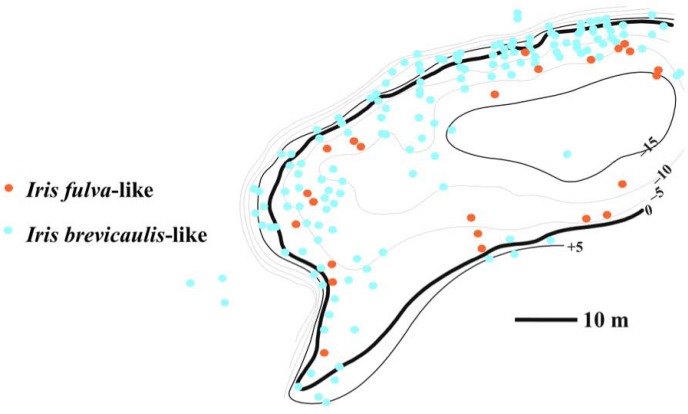
Spatial distribution of Louisiana Iris genotypes in a natural population containing “*I.* *brevicaulis*-like” and “*I. fulva*-like” genotypes. Each circle reflects a single plant. The numbers indicate elevations, with the “0” line indicating the water level of the pond. Negative values reflect flooded areas, and positive values reflect areas above the waterline [19].

The above two observations led Martin *et al.* [31] to conclude that introgression of adaptive alleles would occur in natural hybrid zones under certain environmental conditions. In particular they stated, “While some proportion of this introgression almost certainly involves neutral loci, any selectively advantageous alleles are likely to introgress across the species’ boundaries [32]. In the case of *I. brevicaulis* and *I. fulva* hybrid zones, we predict that the QTL found to promote tolerance to flooded environments are candidates for introgression, since they will presumably be positively selected under flooded conditions.” In this context, Figure 7 illustrates a natural hybrid population between *I. fulva* and *I. brevicaulis* in which ecological-genetic associations suggest the process of asymmetric, adaptive trait introgression. *Iris brevicaulis* genotypes are not adapted to extended, flooded environments [18,31], yet in this population “*I. brevicaulis*-like” plants occur mostly in this type of habitat (Figure 7; [19]). This observation, in the light of previous findings [18,31], supports the inference of adaptive trait transfer from *I. fulva* into *I. brevicaulis* in this natural hybrid population allowing the latter to invade habitats not open to non-introgressed *I. brevicaulis*.

## 7. Conclusions

Evolutionary and ecological studies of Louisiana Irises have confirmed the process of asymmetric introgression. In both experimental and natural hybrid populations, genetic material predominantly moves from *I. fulva* into both *I. brevicaulis* and *I. hexagona*. Furthermore, analyses designed to dissect out the various components of reproductive isolation have determined some of the causal factors – both prezygotic and postzygotic – that contribute to this asymmetry. In particular, gamete competition and viability selection likely contribute to the biased production of zygotes that contain more introgressed *I. brevicaulis* and *I. hexagona*, than introgressed *I. fulva* genotypes. It is also likely that, under certain environmental conditions, the incorporation of *I. fulva* alleles into the other two species is adaptive. Asymmetric introgressive hybridization among the Louisiana Iris species has thus likely affected not only the population genetic structure of hybrid zones, but also to some degree determined the ecological and evolutionary trajectories of the hybrids and introgressed species.
